# Successful management of complex haemorrhagic pericardial cyst with cirrhosis of liver: a case report

**DOI:** 10.1093/icvts/ivac278

**Published:** 2022-11-21

**Authors:** Amy J Lykins, Pankaj Garg, Zachary J Fleissner, Basar Sareyyupoglu

**Affiliations:** Department of Cardiothoracic Surgery, Mayo Clinic, Jacksonville, FL, USA; Department of Cardiothoracic Surgery, Mayo Clinic, Jacksonville, FL, USA; Division of Anesthesia, Mayo Clinic, Jacksonville, FL, USA; Department of Cardiothoracic Surgery, Mayo Clinic, Jacksonville, FL, USA

**Keywords:** Pericardial cyst, Haemorrhage, Calcification, Right heart failure, Angiotensin, Vasoplegia syndrome

## Abstract

Haemorrhagic pericardial cysts are rare and may be complicated by encasement of the heart, right heart failure and, rarely, cardiac cirrhosis. Surgical management of complicated cysts is challenging and has poor outcomes. We report a case of successful surgical management of a complicated pericardial cyst presenting with cardiac cirrhosis and the challenges associated with this condition.

## INTRODUCTION

Pericardial cyst can rarely be complicated by haemorrhage due to internal bleeding or erosion into adjacent structures. However, if goes unnoticed, the cyst may encase the heart resulting in right heart failure and rarely cardiac cirrhosis. Therefore, surgical management of complicated pericardial cyst is challenging [1–3]. We report the first case of successful surgical management of complicated haemorrhagic pericardial cyst with cardiac encasement resulting in cardiac cirrhosis.

## CASE REPORT

**Figure 1:**
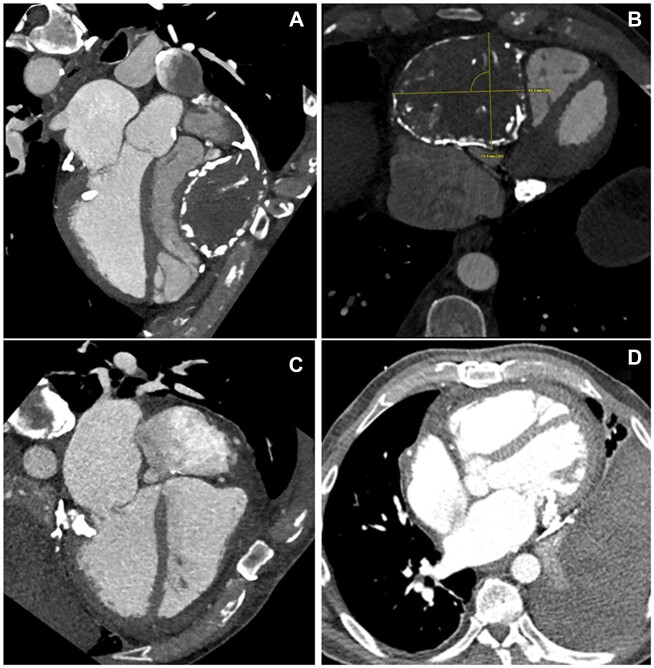
Preoperative CECT chest showing calcified pericardial cyst significantly compressing the tricuspid annulus, right atrium and right ventricle (**A** and **B**). Postoperative CT chest showing the complete removal of the pericardial cyst and relieved right atrial and right ventricle compression (**C** and **D**).

**Figure 2:**
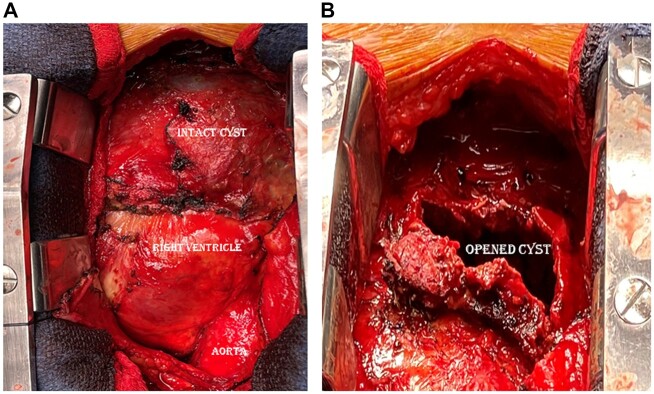
Intraoperative photos of the intact pericardial cyst (**A**) and opened pericardial cyst (**B**).

A 63-year-old man with 20 years history of pericardial cyst presented with lower extremity swelling, worsening dyspnoea and yellowish discolouration of the eyes for 4–5 months. His total bilirubin was 3.9 mg/dl. MRI of the abdomen revealed hepatic cirrhosis without ascites, pericardial or pleural effusion. Contrast-enhanced computed tomography findings are shown in Fig. 1. Echocardiography revealed a left ventricle ejection fraction of 48%, dilated inferior vena cava with <50% respiratory variation, severe tricuspid regurgitation and mitral annular calcification without mitral regurgitation or stenosis. There was an 80% lesion in the mid-right coronary artery on cardiac catheterization. The patient was operated on 2 weeks after the presentation, through a midline sternotomy. The cyst wall was severely calcified and densely adhrent to and encasing the right heart. It contained paste-like material and old clots (Fig. 2). We excised the cyst with blunt and electrocautery-assisted dissection. At places where the cyst was severely calcified, it was partially decalcified before excision with the help of a Sonopet ultrasonic aspirator (Stryker, MI, USA). To limit the cardiopulmonary bypass (CPB) duration, most cyst excision was performed without CPB. Subsequently, CPB was established by aorto-bicaval cannulation and on mild hypothermic cardioplegic arrest, we completed the cyst excision and performed right coronary artery bypass grafting using reversed saphenous vein graft, excision and closure of left atrial appendage and ring annuloplasty of TV. The total CPB time was 2 h and 43 min, and the total myocardial ischaemic time was 1 h and 55 min.

Weaning from CPB was complicated by severe vasoplegic syndrome (VS) despite multiple vasopressors, including nor-epinephrine, vasopressin, methylene blue and hydroxycobalamin. We initiated the angiotensin-II (AT-II) infusion that stabilized the blood pressure and reduced the dose of other vasopressors. The patient was extubated on postoperative day (POD) 4; however, the patient required vasopressors till POD 17. Another complication was excessive mediastinal bleeding. Due to the presence of RV dysfunction, we transfused KCENTRA^®^ (CSL Behring GmbH, Marburg, Hessen, Germany) to limit to transfusion of blood and blood products. The patient was discharged on POD 25 in stable condition. Echocardiography at the time of discharge showed mild RV dysfunction, mild tricuspid regurgitation and normal left ventricle ejection fraction. Contrast-enhanced computed tomography of chest confirmed the complete removal of the cyst with relief of RA and RV compression (Fig. 1). Histopathology of the pericardial cyst showed the predominance of fibrous tissue and calcific debris. Special stains were negative for amyloid and iron deposition. At the last follow-up, 8 months after surgery, the patient was doing well with complete resolution of symptoms and normalization of serum bilirubin.

## DISCUSSION

Pericardial cysts are most commonly congenital in origin; however, they can be acquired due to tuberculosis, trauma, post-cardiac surgery, rheumatism, pericarditis, echinococcosis or patients on chronic haemodialysis. Our case is unique and has several learning points. First, pericardial cyst, even if asymptomatic, may result in the incarceration of the heart and its complications [1]. Therefore, patients with pericardial cyst who are not operated on should be regularly followed with imaging.

Second, ours is the first case report of successful operative treatment of complicated pericardial cyst. Nguyen *et al.* [3] reported a patient with a large calcified pericardial cyst, cardiac cirrhosis and hepatorenal syndrome. Their patient died prior to surgery due to bacterial peritonitis. Our successful outcome was contributed by the absence of advanced cardiac cirrhosis in our patient.

Third, operating on a patient with right heart failure with cardiac cirrhosis on CPB is a surgical challenge. Both these conditions increase the risk of postoperative VS and coagulopathy due to an increase in inflammatory mediators, hepatic synthetic dysfunction and sequestration of platelets in the spleen. Coagulopathy is usually managed by transfusion of blood and blood products which is poorly tolerated by dysfunctional RV. To prevent this vicious cycle, CPB duration should be kept minimum [4] and coagulation factor concentrate should be transfused upfront as we did in our patient.

Fourth, post-cardiac surgery VS is conventionally managed by vasopressors, methylene blue and hydroxycobalamin. AT-II is reserved as a last resort. In recent post hoc analysis of the ATHOS III trial, mortality was reduced with the use of AT-II in patients with post-cardiac surgery VS [5]. Therefore, the threshold to start AT-II should be kept low.

Fifth, calcified cysts usually engrave into the epicardium and myocardium, and it is challenging to excise the cyst without damaging the heart. Again, an ultrasonic aspirator is extremely helpful in these situations as it breaks the calcium without damaging the soft tissue.

In conclusion, surgical intervention is possible for calcified pericardial cysts associated with cardiac cirrhosis. However, surgical planning, minimizing CPB, use of ultrasonic aspirator and pre-emptive management of VS and coagulopathy are critical to a successful outcome.

**Conflict of interest:** none declared.

## Reviewer information

Interactive CardioVascular and Thoracic Surgery thanks the anonymous reviewer(s) for their contribution to the peer review process of this article.
